# Production of Novel Polygalacturonase from *Bacillus paralicheniformis* CBS32 and Application to Depolymerization of Ramie Fiber

**DOI:** 10.3390/polym11091525

**Published:** 2019-09-19

**Authors:** Md. Saifur Rahman, Yoon Seok Choi, Young Kyun Kim, Chulhwan Park, Jin Cheol Yoo

**Affiliations:** 1 Department of Pharmacy, College of Pharmacy, Chosun University, 309, Pilmun-daero, Dong-gu, Gwangju 61452, Korea; saifpharmacist@outlook.com (M.S.R.); ztakeiteasy@gmail.com (Y.S.C.); arirangkyk1114@naver.com (Y.K.K.); 2 Department of Chemical Engineering, Kwangwoon University, 20, Kwangwoon-ro, Nowon-gu, Seoul 01897, Korea

**Keywords:** *Bacillus*, enzymatic depolymerization, polygalacturonase, ramie fiber

## Abstract

Polygalacturonase (EC. 3.2.1.15) is an enzyme that hydrolyzes the alpha-1,4 glycosidic bonds between galacturonic acid. In this study, an alkaline polygalacturonase producer *Bacillus paralicheniformis* CBS32 was isolated from kimchi (conventional Korean fermented food). The 16S rRNA sequence analysis of the isolated strain revealed that it was 99.92% identical to *B. paralicheniformis* KJ 16LBMN01000156. The polygalacturonase from *B. paralicheniformis* CBS32 was named PN32, and the purified PN32 showed a 16.8% yield and a 33-fold purity compared to the crude broth. The molecular mass, 110 kDa, was determined by SDS-PAGE, and the active band was confirmed by zymography analysis. The N-terminal amino acid sequence residues of PN32 were determined to be Gly–Val–Lys–Glu–Val–X–Gln–Thr–Phe. In the sequence comparison, PN32 was suggested as a novel polygalacturonase, since the sequence was not matched with the previous reports. In an application study, enzymatic depolymerization of ramie was performed for fiber degumming, and the result showed that the PN32 had a 28% higher depolymerization compared to the commercial pectinase. Overall, based on the results, PN32 has high potential for industrial applications.

## 1. Introduction

Fibrous biomass has been utilized as feedstock in various bio-industries, and the cell wall components are mainly composed of cellulose, hemicellulose, and lignin. In particular, pectin is contained in the case of dicotyledonous plants [1,2,3,4]. Pectins, highly complex polysaccharides, are categorized as four subclasses such as homogalacturonan (HG), rhamnogalacturonan I (RGI), rhamnogalacturonan II (RGII), and xylogalacturonan (XGA). The backbones of HG, RGII, and XGA are composed of *α*-1,4-linked galacturonic acid residues. However, the RGI is composed of alternating rhamnose and *α*-1,4-linked galacturonic acid residues with side chains containing arabinose and galactose [5,6]. As described above, complexly structured polymers like pectin can be depolymerized by pectinases that are classified into three major types: Pectinesterases, hydrolyzing glycosidic linkages, and cleaving glycosidic linkages. Pectinesterase (E.C. 3.1.1.11), known as pectin methyl hydrolase, catalyzes the de-esterification of pectin into pectate and methanol. This enzyme preferentially acts on a methyl ester group of the galacturonate unit next to the non-esterified galacturonate unit. Depolymerization enzymes of glycosidic linkages are classified into hydrolytic and cleaved forms. Polymethylgalacturonase and polygalacturonase are representative hydrolytic enzymes that depolymerize *α*-1,4-glycoside bonds and have exo and endo forms. The cleavage of pectin or pectic acid by trans-elimination can be actively catalyzed by polymethylegalacturonate lyase and polygalacturonate lyase. Over the last few decades, pectinases have been used in industrial areas for the degumming and retting of fiber, the improving of paper quality, the extraction of juice and oil, the fermentation of coffee and tea, and the treatment of pectic wastewater [6,7,8]. Polygalacturonase (E.C. 3.2.1.15) is probably the most important biocatalyst among the pectin hydrolyzing enzymes and is known to be the most effective enzyme for the pectin hydrolysis [9]. The degumming and retting of fibers are significant processes in the textile industry, and the use of polygalacturonase has attracted much attention due to its minimization of fiber damage and its high efficiency of degumming [10,11].

Ramie, one of the strongest natural fibers, is known for its ability to hold thread shape and reduce wrinkling, and it is generally used to make such products as clothing fabrics, packing materials, canvas, cordages, fishing nets, and filters. However, despite its excellent properties and various applications, it cannot not become a major textile crop, since it has been difficult to obtain high-quality fibers from ramie by conventional retting process [11,12,13]. Mohanty et al. reported the chemical composition of ramie plant as 68–76% cellulose, 13–17% hemicellulose, 1.9% pectin, and 0.6–0.7% lignin [14,15]. Gum is mainly composed of pectin and hemicellulose, which is almost identical to the other report that ramie fiber contains 20–30% gum [12,16]. It is necessary to remove the plant gum remaining between the fibers for most applications. In general, the degumming process has been carried out by chemical treatment using hot alkaline solutions (12–20% NaOH). However, the fibers can be damaged by the chemical reaction, and the process requires a high energy input, but it also produces hazardous waste [13].

In order to solve these disadvantages, the retting process of bast fibers has been developed by using microorganisms. The main purpose of retting is to selectively decompose without damaging the main component (fibers) and to remove impurities such as pectin, lignin, plant gum and hemicellulose. In the early introduction of retting, the fibers were soaked in a mixed culture of various fungal strains such as *Aspergillus*, *Cladosporium*, *Cryptococcus*, *Penicillium* and *Rhodotorula*. However, because the culture solution contained not only pectinase and hemicellulase but also cellulase, the overall yield decreased due to the decomposition of cellulose. Since then, pure bacterial culture has been applied to cellulase-free conditions and has shown higher yields and product quality without loss of fiber than mixed cultures [12,13,17,18].

As an improved technology, Gillespie et al. suggested that the enzymatic retting could be faster and more reproducible than traditional methods, and more uniform and higher quality fibers can be provided to the spinner [19]. In addition, a combined chemical and biological process has been designed for the efficient degumming of ramie bast fibers, and it was reported that an alkaline pretreatment of ramie could accelerate the degumming process. However, there is still no report on the successful and economical application of a biotechnological degumming on an industrial scale [11]. The demand for industrial enzymes for such applications is increasing rapidly; however, research on the development is still a challenging task. It is expected that specific pectinolytic enzymes with more stable and higher activity will be required in near future.

In this study, an enzyme with the highest polygalacturonase activity on the alkaline phase was selected to be utilized in the biological depolymerization of gum. The producer of the enzyme with the highest activity was the *Bacillus* CBS32 strain isolated from kimchi, and the purified polygalacturonase was named as PN32. In addition, the biochemical characterization of PN32 was carried out, and its application for the depolymerization of ramie fibers was evaluated.

## 2. Materials and Methods 

### 2.1. Materials

Pectin (from apple) and pectinase (from *Aspergillus niger*) were purchased from Sigma-Aldrich (St. Louis, MO, USA). The ramie fiber was purchased from a local vendor, Gwangju, South Korea. The Sepharose CL-6B Column was obtained from GE Healthcare Bio-Science AB (Uppsala, Sweden). All analytical grade reagents were used.

### 2.2. Screening, and Identification of the CBS32 Strain

The kimchi, a popular conventional Korean fermented food, samples were collected from different provinces of South Korea. To isolate the bacteria, 1 g of kimchi was mixed with 0.80% NaCl and kept at 37 °C for 24 h. Serial dilutions were done up to 10^−7^, and it was streaked in a Mueller–Hinton agar (MHA) plate to adjust colony forming unit (CFU). In total, seventy-eight isolated strains were stocked in 20% glycerol at −80 °C by appropriately diluting. In the initial screening, 77 strains were streaked on polygalacturonase plates containing 1.5% pectin mixed with 0.5% of 1% tryptone, 0.7% K_2_HPO_4_, yeast extract, 0.01% MgSO_4_.7H_2_O, 0.3% KH_2_HPO_4,_ and 1.5% agar, and they were incubated at 37 °C for 36 h. The iodine-potassium iodide solution (1 g of iodine, 5 g of potassium iodide, and 330 mL of H_2_O) was added to plates of polygalacturonase media containing 2–3 mm long colonies and incubated for 20 min; afterwards, it was washed with 1 M NaCl twice to detect pectin utilization clearance zones. The selected strains from initial screening were cultured in 250 mL Erlenmeyer flasks containing 50 mL of polygalacturonase media at 37 °C for 84 h. Polygalacturonase activity was monitored every 12 h. The maximum polygalacturonase activity was displayed for the CBS32 strain and considered for the current study. The identification of selected CBS32 was performed according to Bergey’s Manual of Systemic Bacteriology and based on morphological and 16S rRNA gene sequence analysis [20].

### 2.3. Protein Measure and Polygalacturonase Activity

Protein concentrations were estimated by the Bradford method using bovine serum albumin as the standard. Polygalacturonase activities were assayed by adding 100 μL of enzyme (diluted in 10 mM Tris–HCl, pH 9.0) to a 100 μL substrate of a 0.75% pectin solution (from apple, Sigma-Aldrich, St. Louis, MO, USA) and kept for 50 min at 60 °C. In the meantime, prepared samples mixture for control were incubated at 4 °C. The reducing sugars released by PN32 were measured by adding 100 μL of a 0.5% 3,5-dinitrosalicylic acid (DNS) solution, as described by Miller [21]. One unit (U) polygalacturonase activity was defined as the amount of enzyme that released 1 µmol of galacturonic acid per min under standard assay.

### 2.4. Production and Purification of PN32

To culture the CBS32 strain, a 2 L Erlenmeyer flask was used for 200 mL of polygalacturonase media at 37 °C with constant shaking at 100 rpm, and polygalacturonase activity was measured every 12 h. The cell-free supernatant was collected at 36 h and mixed with ammonium sulfate (30–80% saturation) and kept at 4 °C overnight with stirring. The precipitation was collected gently by centrifuging for 30 min at 10,000 × *g* and resuspended in a 10 mM Tris–HCl buffer (pH 9.0). The fractions saturated in 30–80% ammonium sulfate were desalted by using an ultra-filtration membrane (30 kDa, Millipore, Burlington, MA, USA), and the proteins of molecular weight (MW) higher than 30 kDa were recovered. The sample was taken for loading into a Sepharose CL-6B column (1.5 × 30 cm) using a 10 mM Tris–HCl buffer (pH 9) system. Fractions were collected at a flow rate of 0.3 mL/min for 3 mL.

### 2.5. Determination of Molecular Weight

The method as described by Laemmli was used to determine the MW of the purified enzyme using 12% (*w*/*v*) sodium dodecyl sulfate-polyacrylamide gel electrophoresis (SDS-PAGE). Protein bands were observed by staining with dye (Coomassie Brilliant Blue R-250, Thermo Fisher Scientific) and followed by destaining with a solution (methanol:Glacial acetic acid:Distilled water = 1:1:8 by vol). The further confirmation of MW was confirmed with zymogram as described by De Sousa, with minor modifications [22].

### 2.6. The N-terminal Sequencing

To determine the amino acid sequences, the purified PN32 was subjected to the Edman degradation method using a Procse Model 492 protein sequencer (Applied Biosystems, Foster, CA, USA) to determine the N-terminal amino acid sequence.

### 2.7. Effect of pH and Temperature on Polygalacturonase Activity

The relative activity of polygalacturonase was determined at various pH levels of 10 mM (pH 2.0–14). The pH stability was measured by incubating enzyme aliquots at 4 °C for 2 and 24 h with respective pH buffers (pH 2.0–14.0). The residual activity was determined under standard assay conditions. To determine the optimum temperature, enzyme samples were incubated at 30–100 °C for 1 h, and the relative activity of polygalacturonase was investigated. The thermal stability of enzyme samples was measured at 60, 70, 80, 90 and 100 °C for 4 h. Aliquots of samples were withdrawn at every 5, 15, 30, 60, 90, and 120 min, and the thermal residual enzyme activity was measured as described earlier.

### 2.8. Effects of Metal Ions and Kinetic Parameters

The effects of metal ions on polygalacturonase activity were examined at 60 °C. The pure PN32 (0.01 mg) was incubated in a 5 mM final concentration of different metal ions. The relative activity of PN32 was measured by comparing it to a control group without metal ions (100%). The Michaelis constant (K_m_), maximum reaction rate (V_max_), and kinetic constant of purified PN32 were measured using the Lineweaver–Burk equation [23], Hanes–Woolf equation [21] and Eadie-Hofstee equation [22], with minor modifications. A constant enzyme concentration (0.01 mg) was incubated with different concentrations of pectin (0.625–10 mg/mL) under the standard assay conditions. 

### 2.9. Enzymatic Depolymerization of Ramie Fiber

Untreated ramie fiber was considered as a control following an initial wash with a 10 mM Tris–HCl buffer (pH 9.0) and then dried completely. The ramie fiber samples were treated with an enzyme solution (100 U/mL) at 40 °C for 2.5 h. Afterward, depredated ramie fibers were analyzed by scanning electron microscopy (SEM) with 400× and 800× magnifications [24]. The properties of ramie fibers were measured according to the method as described by Guo et al. [25]. The gum loss of the fibers was calculated as follows:

Gum loss (%) = (*W*_0_ − *W*_1_)/(*W*_0_ × *W*_2_) ×100 (1)

where *W*_0_ and *W*_1_ are the weights of the dry ramie fibers before and after degumming, respectively. *W*_2_ is the gum content of the ramie fibers before degumming.

## 3. Results

### 3.1. Strain Identification, and Enzyme Production

**Figure 1:**
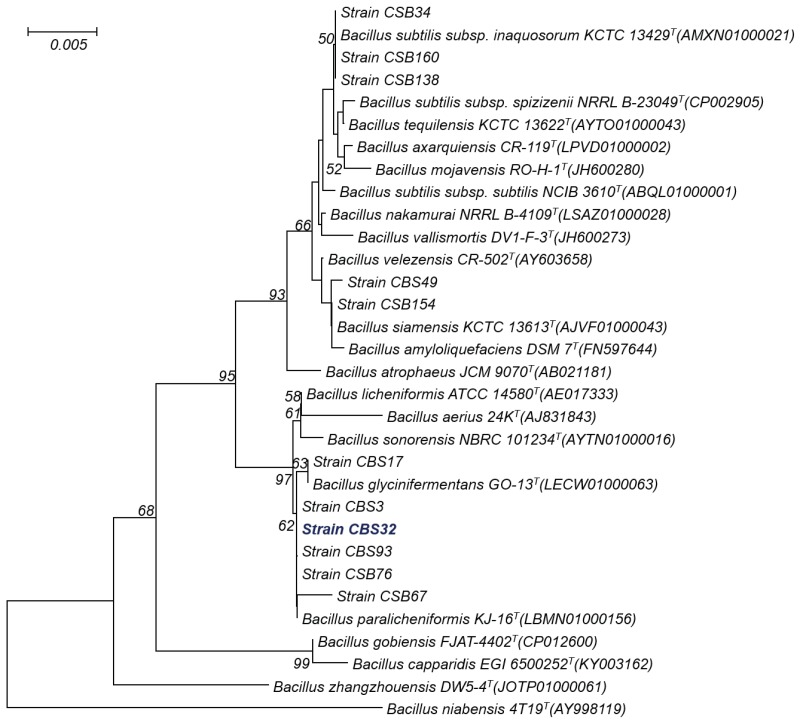
Neighbor-joining based phylogenetic on nearly complete 16S rRNA gene sequences showing relationships among CBS32 and closely related taxa of the genus *Bacillus*.

The CBS32 strain showed the highest polygalacturonase activity among the 80 cultured strains screened and was selected for further study. A phylogenetic tree developed from the 16S rRNA sequence and analysis identified the CBS32 as 99.92% identical to *Bacillus paralicheniformis* (Accession: 16LBMN01000156) (Figure 1). The *Bacillus paralicheniformis* CBS32 strain was submitted at the Korean collection for type culture (KCTC), which belongs to the World Data Centre for Microorganisms (WDCM). The accession number was denoted as KCTC18677P. The highest production of PN32 was achieved in 36 h in optimized polygalacturonase media at 37 °C.

### 3.2. Purification of PN32

**Figure 2:**
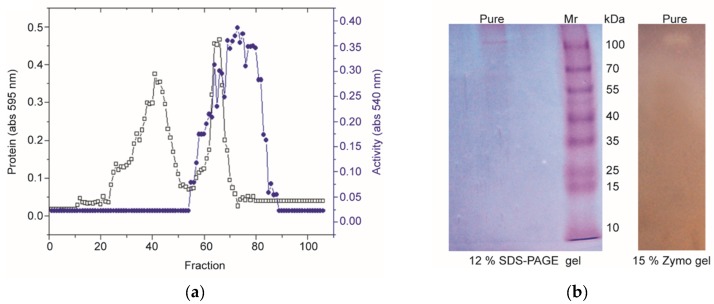
(**a**) The elution profile of PN32 from the Sepharose CL 6B permeation column (1.5 × 30 cm); (**b**) SDS-PAGE and zymography of purified PN32. Lane M: Protein molecular weight marker; lane pure: Purified PN32 after chromatographic separation by the Sepharose CL 6B column.

The polygalacturonase media were used for the production of polygalacturonase (PN32) from *Bacillus paralicheniformis* CBS32. The purification of PN32 from the 36 h culture supernatant (30–80% ammonium sulfate saturation) of CBS32 is summarized in Table 1. A homogeneity column procedure was performed to purify PN32 (Figure 2a), resulting in 33-fold purification and 16.8% activity recovery. The purified PN32 in SDS-PAGE showed the single band of molecular weight to be 110 kDa, and this was confirmed by the activity staining of the gel (zymography), as shown in Figure 2b. The purified PN32 showed a specific activity of 4053.8 U/mg with a yield of 16.8%.

### 3.3. N-terminal Amino Acid Sequence Analysis

**Figure 3:**
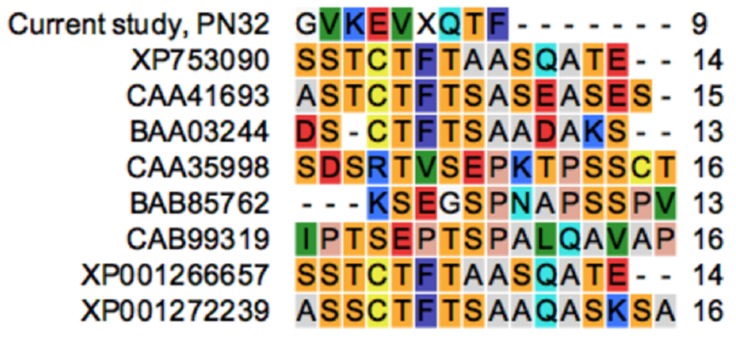
Comparison of the N-terminal amino acid sequence of existing polygalacturonase and PN32.

The N-terminal amino acid sequence residues of PN32 were determined as Gly–Val–Lys–Glu–Val–X–Gln–Thr–Phe. Sequence analyses and a BLAST search against GenBank suggested that PN32 possesses an entirely novel amino acid sequence (Figure 3).

### 3.4. Effects of pH, Temperature, and Metal Ions on PN32

**Figure 4:**
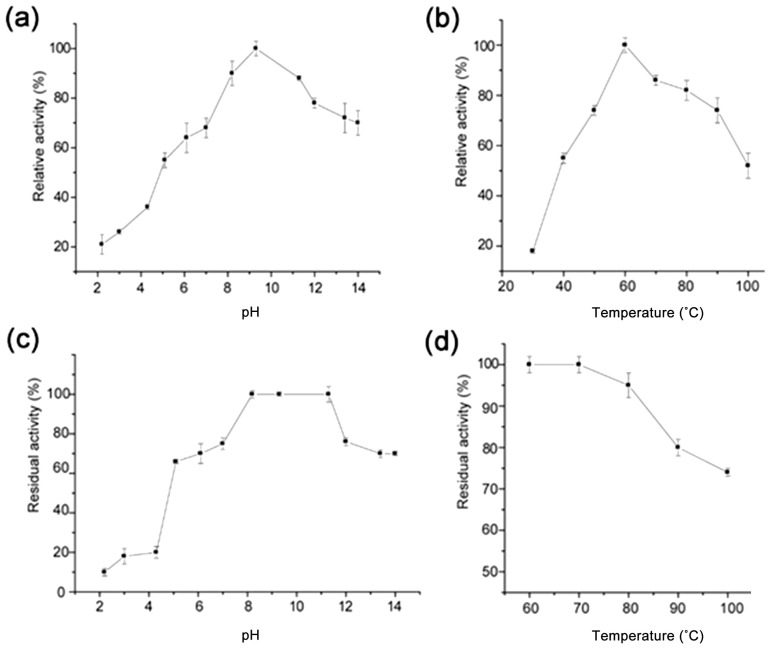
(**a**) Optimum pH, (**b**) optimum temperature, (**c**) pH stability, and (**d**) thermal stability of purified PN32. To determine optimum pH, the reaction was carried out at 60 °C using different pH buffers (2–13.6). The pH stability of PN32 was performed at 4 °C for 24 h, and the relative activity was evaluated under standard assay conditions. To determine optimum temperature, the reaction was performed in an optimum pH (12.2) at different temperatures (30–100 °C), whereas the thermal stability was measured in the temperature range of 60–100 °C for 1 h. Each value is represented as the mean (n = 3).

The pH and temperature left notable effects on the activity and stability of enzymes, which is not unusual for PN32. The optimum pectinase can be broadly classified into acidic and alkaline enzymic based on the pH requirement. Alkaline pectinases are used for the degumming of bast ramie fibers and the pretreatment of wastewater for the food processing industry. The effects of pH and temperature on the PN32 activities and stabilities compared to alkaline pectinase from *Bacillus* sources are shown in Table 2 [12,23,25,26,27,28,29,30]. The effect of pH on PN32 activity was investigated at an optimized temperature at 60 °C in the buffer (10 mM Tris–HCl, pH 9.0). As shown in Figure 4, the PN32 remained active over a wide-ranging pH, exhibiting over 50% of activity between pH 9.0 and 12.3, with an optimum pH of 9.0; this corresponded with values (pH 10.0) reported form Kapoor et al. (2001) [12]. PN32 had the highest relative activity at 60 °C and maintained ~80% stability up to 90 °C. In general, metal ions are known to significantly affect the activity of enzymes, which play an important role in modulating the conformational flexibility of ions. The effects of various metal ions on PN32 activities are presented in Table 3. The results showed that the polygalacturonase activity of PN32 did not show a significant improvement by the addition of metal ions, unlike what has been previously reported [20,21,22,23,29,30,31]. PN32 activity with K^+^ (105.3 ± 4.0%), Mg^2+^ (105.2 ± 5.0%), Mn^2+^ (103.6 ± 5.0%), Ni^2+^ (102.4 ± 0.5%), and Zn^2+^ (101.3 ± 0.9%) was similar to the control. On the other hand, in the case of specific metal ions, the enzyme activity was lower than that of the control group (100%) due to inhibition; Ca^2+^ (82.7 ± 1.0%), Cu^2+^ (85.4 ± 1.2%), and Na^+^ (90.1 ± 0.5%).

### 3.5. Kinetics Parameter of PN32

The kinetics parameter, *K*_m_m and *V_max_* of PN32 were calculated by linear (Lineweaver–Burk plot) and non-linear regression (Hanes–Woolf plot and Eadie–Hofstee plot) methods, and the result of each plot are shown in Supplement File (Figure S1: Lineweaver–Burk Plot, Figure S2: Hanes–Woolf Plot and Figure S3: Eadie–Hofstee Plot). The Lineweaver–Burk plot is still used as an effective tool to determine the type of enzyme inhibition (competitive, non-competitive and uncompetitive inhibitors). However, Lineweaver–Burk plot (double reciprocal plot) had a small error in the measurement distorts the value of the data, making it difficult to determine enzyme kinetics parameters. Thus, the non-linear regression (alternative linear forms) of the Michaelis–Menten equation, such as the Hanes–Woolf plot or the Eadie–Hofstee plot, are commonly used for parameter calculations. An enzyme assay was carried out at optimal conditions with varying pectin concentrations (0.625–10 mg/mL) and a constant enzyme concentration (0.01 mg). Table 4 shows the calculation results of *K*_m_ and *V*_max_ using different methods of plotting. Based on the Lineweaver–Burk, Hanes–Woolf and Eadie–Hofstee plots, the determined *K*_m_ for PN32 was 0.021 ± 0.0020, 0.043 ± 0.0064 and 0.021 ± 0.0019 mg/mL, respectively, and the maximum reaction rate (*V*_max_) was achieved about 410.43 ± 1.80, 444.03 ± 9.34 and 413.60 ± 1.08 U/mg, respectively.

### 3.6. Enzymatic Depolymerization of Ramie Fiber by PN32

**Figure 5:**
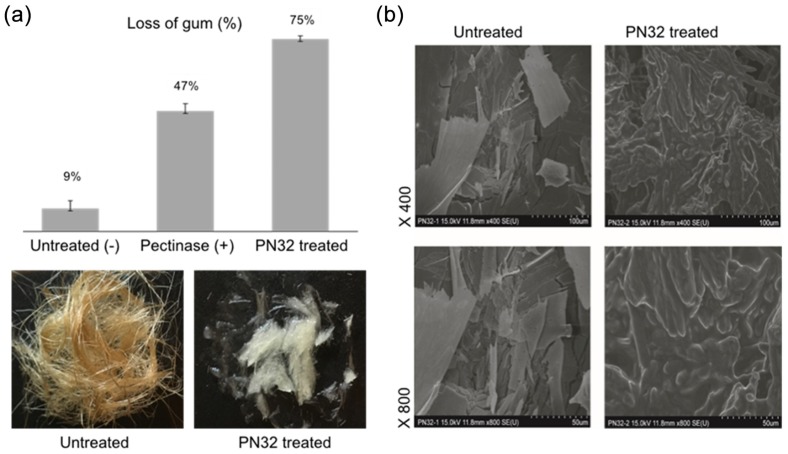
(**a**) Gum loss of the ramie fibers after 2 h treatment by polygalacturonase. The untreated (negative control) means that only a buffer was used, and the positive control means that treatment was done by commercial pectinase (from *Aspergillus niger*, Sigma-Aldrich, St. Louis, MO, USA). Ramie fibers were untreated and PN32-treated (100 U/mg). (**b**) Scanning electron micrographs of ramie fiber degradation by PN32. From left: Control and PN32-treated (100 U/mg). From top: Images at various magnifications (400× and 800×).

Numerous strains with pectinase producing and degumming abilities were isolated in our laboratory over the past five years. Pilot studies showed that the PN32 of the *Bacillus paralicheniformis* CBS32 strain had good potential for degumming ramie fibers. Figure 5a shows that the percentage of degumming with the PN32 was 75% compared to 47% of the commercial pectinase-treated control for 2 h, whereas, the untreated negative control had only 9% of gum loss. The depolymerization of ramie fiber was notably higher when treated with PN32 compared to the control groups. To understand the potential of PN32 in the industry, structural changes in ramie textile by enzymatic hydrolysis were observed using scanning electron microscopy (SEM) (Hitachi SU-70, Hitachi, Japan) (Figure 5b).

## 4. Discussion

Most of the alkaline pectinolytic enzymes were determined as alkaline endo-pectinolytic enzymes, as they demonstrated not only properties of alkaline pectinolytic activity but also showed alkaline endo-pectinase activity. Several earlier pectinase studies have shown that the molecular weight of exo-polygalacturonase is approximate 115 kDa, as estimated by SDS-PAGE [29]. The specific activity values may not necessarily agree with pectinolytic enzymes from different sources due to a high amount of inter-laboratory variability and different mechanisms of alkaline pectinolytic enzymes [31]. These results are consistent with those reported in the recent past for alkaline pectinolytic enzymes obtained from *Bacillus subtilis* ZGL14 [30]. The stable active pectinase under alkaline conditions is commercially crucial for textile and paper industries. However, the utilization of alkaline pectinase for the industrial applications has recently drawn attention [32]. Various studies have reported the effects of various alkaline pectinase sources with metal ions. For example, Khan et al. (2018) reported that the polygalacturonase from *Bacillus paralicheniformis* was activated by Mg^2+^, Zn^2+^, and K^+^ ions but inhibited by Cu^2+^, Ca^2+^ and Na^+^ ions [23]. Contrary to our expectations, purified PN32 was not activated by metal ions. Interestingly, the results of enzyme inhibition were strongly consistent with the report from Khan et al. (2018). The K_m_ value of PN32 (0.21 mg/mL) was lower than other pectinases from *Bacillus* (1.891 mg/mL) [33]. The lower K_m_ value indicated a higher affinity for the substrate. This biochemical characterization of alkaline pectinase is crucial information for industrial applications.

The enzymatic depolymerization of ramie fiber was carried out using alkaline PN32 to investigate application potential. Significantly physical changes of the ramie fibers were also observed using SEM. The PN32-treated samples showed a decomposed and smoother surface, suggesting that PN32 removed the gum-like material and could also provide better degumming effects (Figure 5a) compared to previous bio-degumming reports of the alkaline pectinolytic enzyme PEL168P from *Bacillus subtilis* [34]. This result proves that PN32 breaks down strong natural ramie fibers, which suggests that PN32 may break down not only fiber pectin but also other celluloses and lignin. Therefore, PN32 is effective in industrial fiber degumming, and it can potentially be used as a biomass-degradation agent in the bioconversion process of biorefinery, which uses biomass as a raw material. To conclude our study, a novel strong alkaline polygalacturonase PN32 was produced from *Bacillus paralicheniformis* CBS32, and this showed the significant degumming capability of ramie fiber. In addition, its alkaline characteristics demonstrate that PN32 is suitable for degumming processes and industrial applications. The complete amino acid sequence of this PN32 enzyme and the full catalytic efficiency of PN32 (kcat/Km) must be determined in our further structure-activity studies. However, we believe that this study enhances the research resources of degumming enzymes and pectinase studies regarding microbe-produced pectinases and commercial applications.

## Figures and Tables

**Table 1:** Purification summary.

Purification Step	Total Vol. (mL)	Total Protein (mg)	Total Activity (U)	Specific Activity (U/mg)	Yield (%)	Purity Fold
**Crude Broth**	800	188	23,112	123	100.0	1.0
**(NH_4_)_2_SO_4_**	15	21	16,254	774	70.3	6.3
**Sepharose CL 6B**	18	0.96	3892	4054	16.8	33.0

**Table 2:** Comparison of PN32 to similar alkaline pectinase from *Bacillus* strains.

*Bacillus* Strains	Type	pH	Temp. (°C)	Ref.
*Bacillus* sp. MG–cp–2	Polygalacturonase	10.0	60	[12]
*Bacillus paralicheniformis* CBS3	Polygalacturonase	9.1	60	[23]
*Bacillus* sp. Y1	Pectate lyase	8.5	50	[25]
*Bacillus clausii* KSM–K16	Pectate lyase	10.5	50–55	[26]
*Bacillus subtilis* PEL168	Pectate lyase	9.5	50	[26]
*Bacillus pumilus* DT7	Polygalacturonase	8.0	45	[27]
*Bacillus subtilis*	Polygalacturonase	9.5	65	[27]
*Bacillus* sp. NT–33	Polygalacturonase	10.5	75	[28]
*Paenibacillus polymyxa* *(Bacillus polymyxa)*	Polygalacturonase	8.4–9.4	60	[28]
*Bacillus stearothermophilus*	Pectate lyase	9.0	70	[28]
*Bacillus pumilus*	Pectate lyase	8.0–8.5	60	[28]
*Bacillus* sp. KSM–P576	Polygalacturonase	8.0	55	[29]
*Bacillus subtilis* ZGL14	Polygalacturonase	8.6	50	[30]
*Bacillus paralicheniformis* CBS32	Polygalacturonase	9.0	60	This study

**Table 3:** Metal ion effects.

Metal Ions	Concentration (mM)	Relative Activity (%) ^a^
Ca^2+^	5	82.7 ± 1.0
Mg^2+^	5	105.2 ± 5.0
Cu^2+^	5	85.4 ± 1.2
Ni^2+^	5	102.4 ± 0.5
Zn^2+^	5	101.3 ± 0.9
K^+^	5	105.3 ± 4.0
Mn^2+^	5	103.6 ± 5.0
Na^+^	5	90.1 ± 0.5
Control	-	100.0

^a^ The results presented are the averages of three (*n* = 3) separate determinations ± standard deviation.

**Table 4:** Kinetic parameters for PN32 by different plots.

Plot Method	*K*_m_ (mg/mL)	*V*_max_ (U/mg)
Lineweaver–Burk plot	0.021 ± 0.0020	410.43 ± 1.80
Hanes–Woolf plot	0.043 ± 0.0064	444.03 ± 9.34
Eadie–Hofstee plot	0.021 ± 0.0019	413.60 ± 1.08

## Supplementary Materials

The following are available online at https://www.mdpi.com/2073-4360/11/9/1525/s1, Figure S1: Lineweaver–Burk Plot, Figure S2: Hanes–Woolf Plot, Figure S3: Eadie–Hofstee Plot.
