# Supplementary figures and images for: Production of Novel Polygalacturonase from Bacillus paralicheniformis CBS32 and Application to Depolymerization of Ramie Fiber
Source: Polymers (Basel). 2019 Sep 19;11(9):1525. doi: 10.3390/polym11091525 (PMC6780255; doi:10.3390/polym11091525)

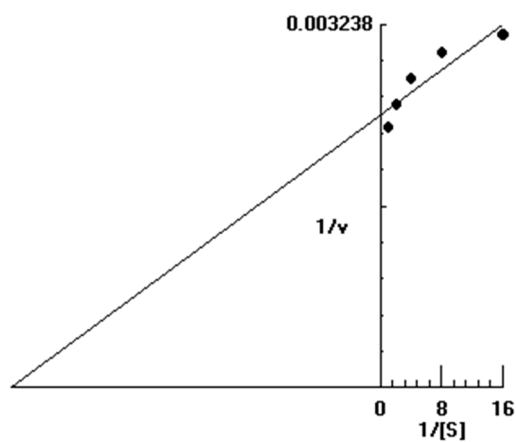

Figure S1. Lineweaver–Burk Plot.

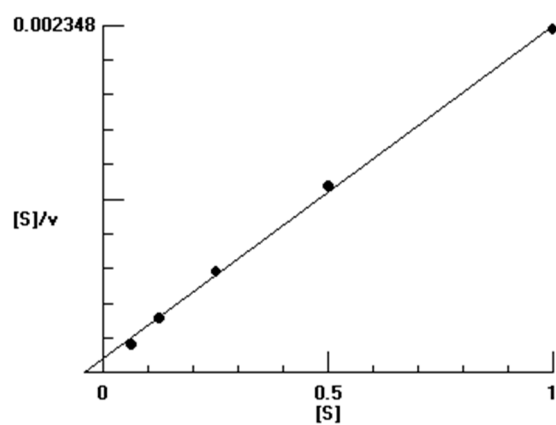

Figure S2. Hanes–Woolf Plot.

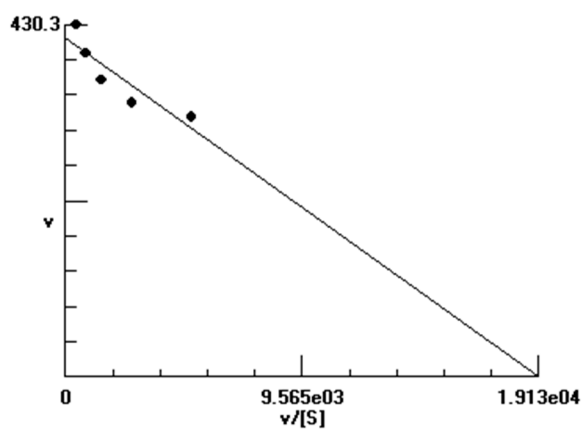

Figure S3. Eadie–Hofstee Plot.

Supplement: Supplementary file 1 [file polymers-11-01525-s001.pdf]
